# Are We Getting High Cause the Thrill is Gone?

**Published:** 2023-12-08

**Authors:** Kenneth Blum, Thomas Mclaughlin, Mark S. Gold, Marjorie C. Gondre-Lewis, Panayotis K. Thanos, Igor Elman, David Baron, Abdalla Bowirrat, Debamyla Barh, Jag Khalsa, Colin Hanna, Nicole Jafari, Foojan Zeine, Eric R. Braverman, Catherine Dennen, Milan T. Makale, Miles Makale, Keerthy Sunder, Kevin T. Murphy, Rajendra D. Badgaiyan

**Affiliations:** 1 The Kenneth Blum Behavioral and Neurogenetic Institute, LLC., Austin, TX, USA; 2 Center for Sports, Exercise, Psychiatry, Western University Health Sciences, Pomona, CA, USA; 3 Institute of Psychology, ELTE Eötvös Loránd University, Budapest, Hungary; 4 Department of Molecular Biology and Adelson School of Medicine, Ariel University, Ariel, Israel; 5 The Sunder Foundation, Palm Springs, CA, USA; 6 Department of Psychiatry, University of Vermont School of Medicine, Burlington, VY, USA; 7 Department of Psychiatry, Wright University, Boonshoff School of Medicine, Dayton, OH, USA; 8 Division of Personalized Medicine, Cross-Cultural Research and Educational Institute, San Clemente, CA, USA; 9 Centre for Genomics and Applied Gene Technology, Institute of Integrative Omics and Applied Biotechnology (IIOAB), Nonakuri, Purba Medinipur, West Bengal, India; 10 Department of Psychiatry, Washington University School of Medicine, St. Louis, MO, USA; 11 Department of Anatomy, Howard University College of Medicine, Washington D.C., USA; 12 Behavioral Neuropharmacology and Neuroimaging Laboratory on Addiction, Research Institute on Addictions, Department of Pharmacology and Toxicology, Jacobs School of Medicine and Biomedical Sciences, University of Buffalo, Buffalo, NY, USA; 13 Cambridge Health Alliance, Harvard Medical School, Cambridge, MA, USA; 14 Division of Therapeutics and Medical Consequences, Medical Consequences of Drug Abuse and Infections Branch, NIDA-NIH, Special Volunteer, Gaithersburg, MD, USA; 15 Department of Applied Clinical Psychology, The Chicago School of Professional Psychology, Los Angeles, CA, USA; 16 Department of Health Science, California State University at Long Beach, Long Beach, CA, USA; 17 Awareness Integration Institute, San Clemente, CA, USA; 18 Department of Family Medicine, Jefferson Health Northeast, Philadelphia, PA, USA; 19 Department of Radiation Medicine and Applied Sciences, UC San Diego, La Jolla, CA, USA; 20 Department of Psychology, UC San Diego, La Jolla, CA, USA; 21 Department of Psychiatry, University of California Riverside, Riverside, CA, USA; 22 Department of Radiation Oncology, University of California, San Diego, La Jolla, USA; 23 Department of Psychiatry, Case University, School of Medicine, Cleveland, OH, USA

**Keywords:** Reward brain circuitry, Thrill is gone, Dopamine, Neurotransmitters, Functional connectivity, Genetic addiction risk severity, Pro-dopamine regulation

## Abstract

In the USA alone, opioid use disorder (OUD) affects approximately 27 million people. While the number of prescriptions may be declining due to increased CDC guidance and prescriber education, fatalities due to fentanyl-laced street heroin are still rising. Our laboratory has extended the overall concept of both substance and non-substance addictive behaviors, calling it “Reward Deficiency Syndrome (RDS).” Who are its victims, and how do we get this unwanted disorder? Is RDS caused by genes (Nature), environment (Neuro-epigenetics, Nurture), or both? Recent research identifies resting-state functional connectivity in the brain reward circuitry as a crucial factor. Analogously, it is of importance to acknowledge that the cumulative discharge of dopamine, governed by the nucleus accumbens (NAc) and modulated by an array of additional neurotransmitters, constitutes a cornerstone of an individual’s overall well-being. Neuroimaging reveals that high-risk individuals exhibit a blunted response to stimuli, potentially due to DNA polymorphisms or epigenetic alterations. This discovery has given rise to the idea of a diminished ‘thrill,’ though we must consider whether this ‘thrill’ may have been absent from birth due to high-risk genetic predispositions for addiction. This article reviews this issue and suggests the general concept of the importance of “induction of dopamine homeostasis.” We suggest coupling a validated genetic assessment (e.g., GARS) with pro-dopamine regulation (KB220) as one possible frontline modality in place of prescribing potent addictive opioids for OUD except for short time harm reduction. Could gene editing offer a ‘cure’ for this undesirable genetic modification at birth, influenced by the environment and carried over generations, leading to impaired dopamine and other neurotransmitter imbalances, as seen in RDS? Through dedicated global scientific exploration, we hope for a future where individuals are liberated from pain and disease, achieving an optimal state of well-being akin to the proverbial ‘Garden of Eden’.

## Preamble

This article represents an enormous amount of work of at least a 60-year sojourn of the first author as well an enormous contribution to the literature in both animal addiction models and clinical science by experts in the field of addiction psychiatry and neuroscience. The co-authors consist of scholarly individuals representing multi-disciplines globally. While there could be some scientific differences there is general agreement by all the co-authors. As a group we applaud the ongoing important work of both NIDA and NIAAA in their quest to find answers to these perplexing questions, especially as to how we could come together and help solve the RDS conundrum.

## Introduction

“Enormous possibilities for health and creativity are held captive by your likes and dislikes. Inspecting your desires and attachments to food [other substances] and making choices intuitively with discrimination will make your spiritual practice and every other relationship more rewarding”.

– Leonard Perlmutter

In this narrative review utilizing PUBMED primarily as the literature search engine we have carefully attempted to recapitulate the existing field related to RDS and generally for reward deficiency.

What happens when the thrill is gone? In fact, what if the thrill was never there to begin with? Dysfunctional brain dopamine caused by RDS - is that when you might just want to get high?

The sun is setting on a balmy Saturday night in New York City, and 14-year-old Gerry is all alone sitting on his family’s balcony overlooking the Hudson River being lit up by the mysterious NY Skyline -a wonderful sight! Talking to his friend Pete, living in the same high rise, no novice when it comes to getting high on weed, who is encouraging Gerry to eat that THC delta 9 gummy that Pete gave him in the morning when Gerry angerly said that he was grounded for two weeks because he lied to his mother about a school project. Gerry’s mother Sue, has been married three times whereby Gerry’s biological father died in an auto accident while driving under the influence of alcohol, weed and cocaine. Sue warned Gerry about this since he could remember as young boy. His 19-year-old sister Barbara is facing DUI charges and, on her way, to rehab. With all this negative information for some unknown reason Gerry for a long time now wanted to get out of his own skin knowing that something was missing. Peter shouted, **“Just take it already”!**

This is now quite a common scenario especially in the good old USA with over one million people fatedly succumbing to powerful opioids. Nearly 27 million people have an OUD according to the 2016 Global Burden of Disease study, and it is still rising as of 2023, most of which occur in the US where opioids are a common class of medication used to treat acute and chronic pain [1]. In 2016 alone, more than 60 million patients had at least one prescription for opioids filled or refilled [1]. However, based on CDC guidelines of only 7 days for acute pain, the number of prescriptions may be declining but fatalities due to fentanyl laced street heroin, for example, are still rising.

Is there a biological and possible genetic answer to why there are millions now in treatment for drug abuse? Do we just blame the Sackler Family for pushing Oxycontin on every street corner in America or do we really want some answers?

Since 1995, a theory has been advanced with respect to the genetic alterations concerning the structure and functioning of the reward system of the brain. This theory, which purports to explain deviant motivations, subsumes a variety of traditional psychiatric diagnoses, including Spectrum Disorders as well as behavioral and substance addictions. It is known as “RDS” and deals with deranged, motivational processes related to the brain’s reward system [2]. Insights afforded by this theory provide a better understanding of the motives of, for example, adolescent drug seeking behaviors than the standalone psychological explanations currently in vogue [3]. So, what is this thing we call RDS? Who are its victims and how do we get this unwanted disorder? Is it our genes (Nature) or caused by our environment that has scientifically referred to neuroepigenetics (Nurture) or a combination of both starting in the mother’s womb [4]. If so, can we identify it at birth or thereafter or would it be better to just stay blind to its etiological root across 8 billion *Homo sapiens* inhabiting the globe we call Earth. It has been estimated that at least 800,000 million carry the DNA tread that causes RDS. In fact, as far back as 2015, the estimated prevalence among the adult population was 18.4% for heavy episodic alcohol use (in the past 30 days); 15.2% for daily tobacco smoking; and 3.8, 0.77, 0.37 and 0.35% for past-year cannabis, amphetamine, opioid and cocaine use, respectively. European regions had the highest prevalence of heavy episodic alcohol use and daily tobacco use. The age-standardized prevalence of alcohol dependence was 843.2 per 100 000 people; for cannabis, opioids, amphetamines, and cocaine dependence it was 259.3, 220.4, 86.0 and 52.5 per 100 000 people, respectively [5]. The percentages have not significantly changed in 2022 and possibly in 2023.

You may also ask how so many people could have this behavioral anomaly and why is it such a secret to the nonscientific community?

**Figure 1:**
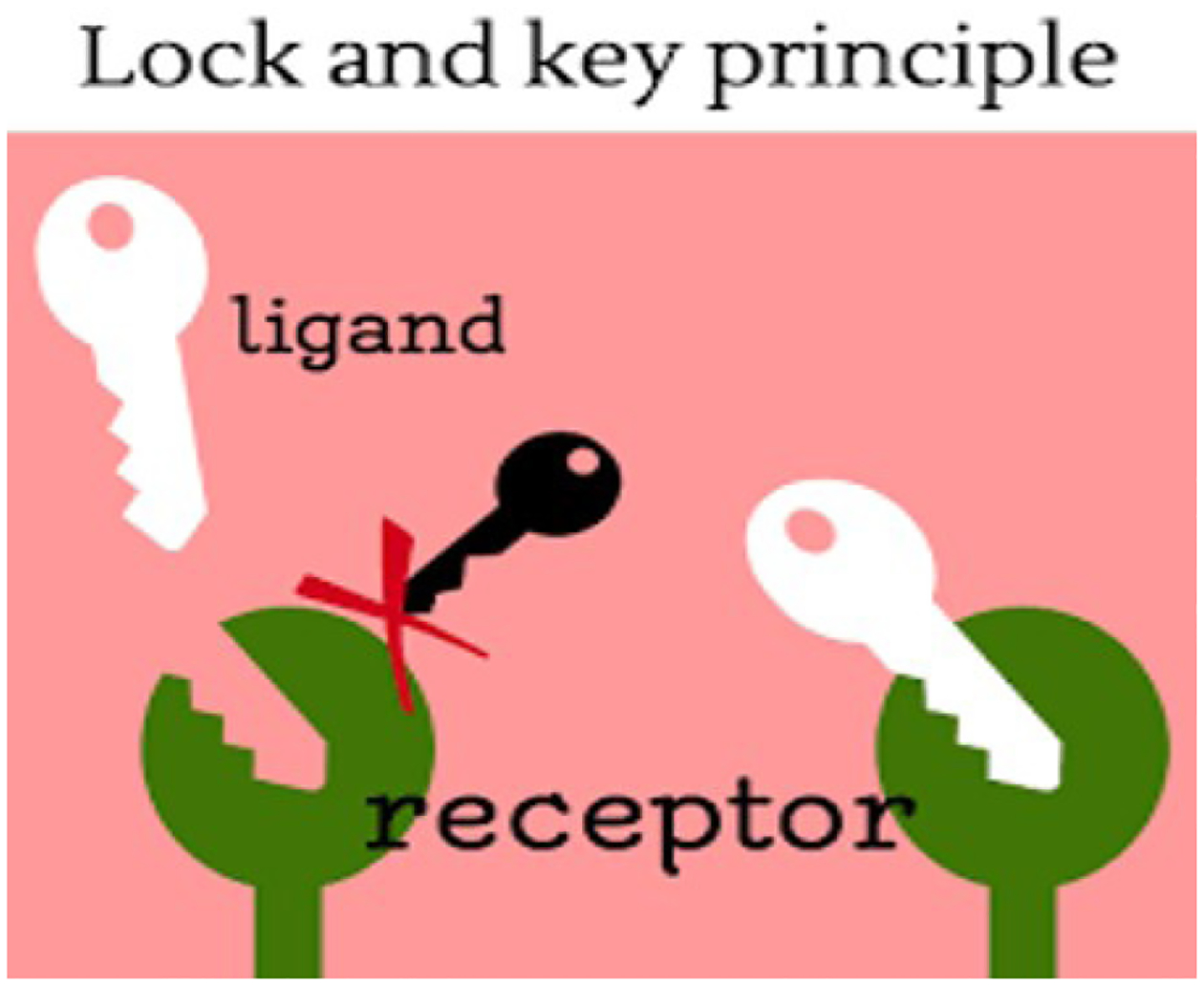
Illustration of the lock and key principle (standard free stock internet).

A simple biogenetic response is that RDS could have evolutionary survival importance, and as such not considered by some a disease but an evolutionary adaptation. Of real interest when we imbibe or act on whatever it is we are addicted to (Substances or Behaviors), the brain systems that “light up” include an especially important molecule called dopamine. While dopamine and it’s accompanied receptors (Protein sites whereby dopamine like a key fitting into a lock called receptors) [Figure 1] is found throughout the brain at specific regions, specially at the “reward” site of the brain called the nucleus accumbens (NAc) and its neighbors in the prefrontal cortex and motor system [6]. In addition, a brain system involved in everything from addiction to autism appears to have evolved differently in humans than in apes, as reported by a large re-search team in the journal *Science* [7].

**Figure 2:**
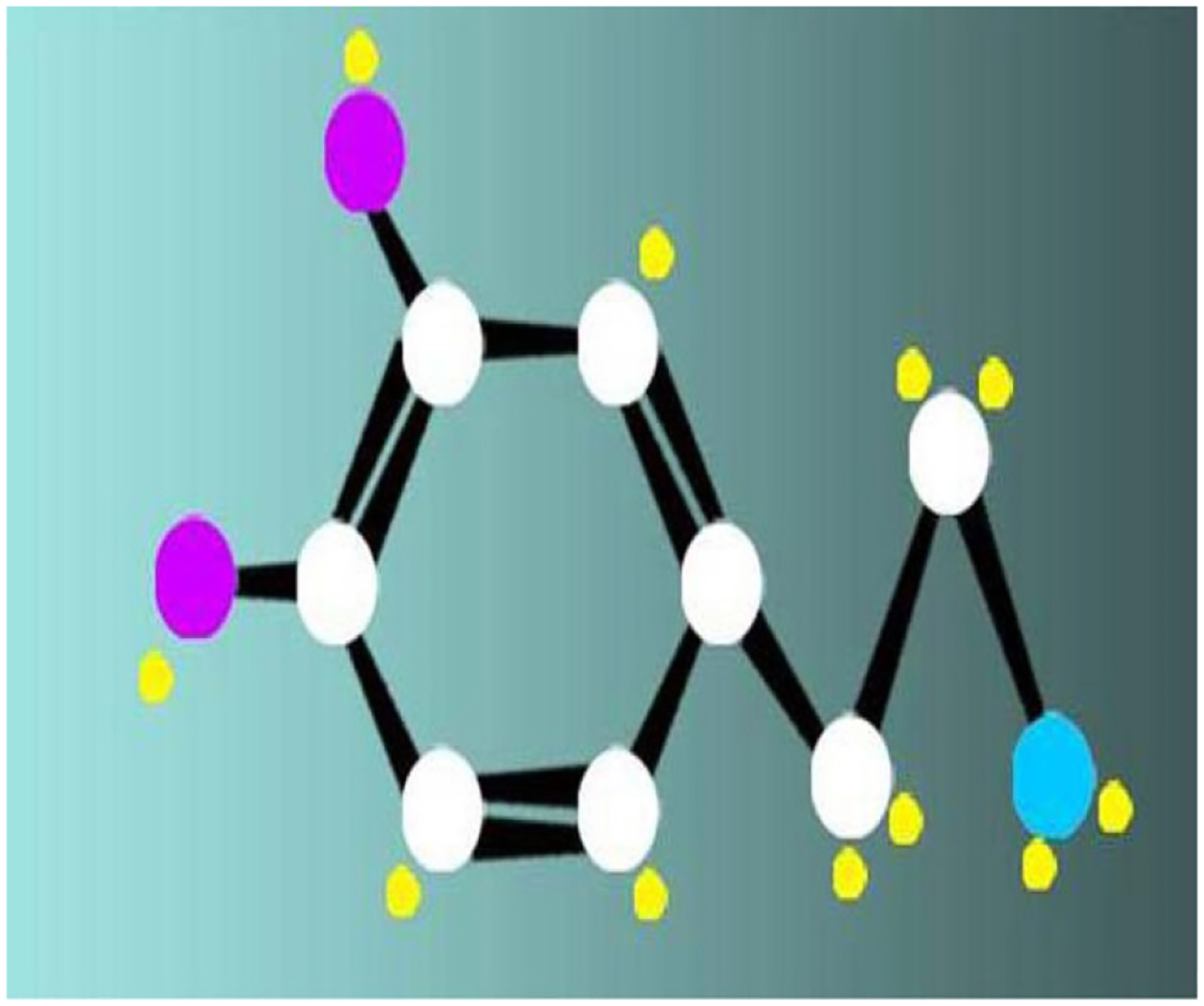
Dopamine chemical structure. The chemical structure of the compound dopamine, the happiness molecule is C_8_H_11_NO_2_. Dopamine affects the brain processes that control emotional responses and ability to experience pleasure, desire, and motivation [26].

The scientific story of RDS begins with the seminal study by Kenneth Blum and Ernest Noble in 1990 when they reported in JAMA that the team discovered the first genetic variant to associate with severe alcoholism [8]. While at that time gene research was in infancy, scientists were perplexed because this was the first study to offer a clue regarding addictive behaviors. While there were other known dopamine receptors (now 9) the original prize goes to the D2 receptor. While the D2 receptor is involved in many behaviors such as drug seeking (Alcohol, marijuana, cocaine, nicotine, and opioids) it is also involved in behavioral addictions as well such as gambling, eating disorders (Anorexia nervosa, bulimia, binge eating, hoarding, etc.) including pleasure, attention, motor control, cognition, and even motivation affecting a motivational syndrome as coined by Dr. David Smith of the Haight Ashbury in the late 70s [9–22]. It is known now that D2 receptor activation by dopamine acts as a filter to offer some “no go” control of over-seeking either drugs or let us say gaming behaviors [23]. Yes, there are other dopamine genes such as the D1 that is indeed the “go” gene [24, 25]. So, it is the n the balance or “homeostasis” that allows for our control not to over imbibe. Is that the reason at least 100,000 million people in America just cannot say no to one or two drinks or where legalized one or two hits of pot- IS IT DOPAMAINE? (Figure 2).

The simple answer is it is not only dopamine, instead there are over 100 neurotransmitters in the brain, as well as many second messengers, and at least 9 major neurotransmitters (including Orexin) that influence the net release of dopamine at the NAc. So, what happens when the D2 receptor population is not normal (e.g., too many or too few)? The common denominator underlying both chemical and behavioral addictions also contributes to compulsive disorders such as obsessive-compulsive disorder (OCD) as well as eating disorders, PTSD, ADD and certain anger disorders. It also overlaps with and influences chronic pain [27, 28]. “Reward Deficiency,” a term first coined by Kenneth Blum in 1995 [8] as a syndrome, having 1,545 ( December 8, 2023) articles published in the scientific literature and the syndrome RDS now with 253 listed articles, is also featured in SAGE Encyclopedia of Abnormal and Clinical Psychology [29] can be defined as: *“A brain reward genetic dissatisfaction or impairment that results in aberrant pleasure-seeking behavior that includes drugs, excessive, food, sex, gaming/gambling and other behaviors.”*

**Figure 3:**
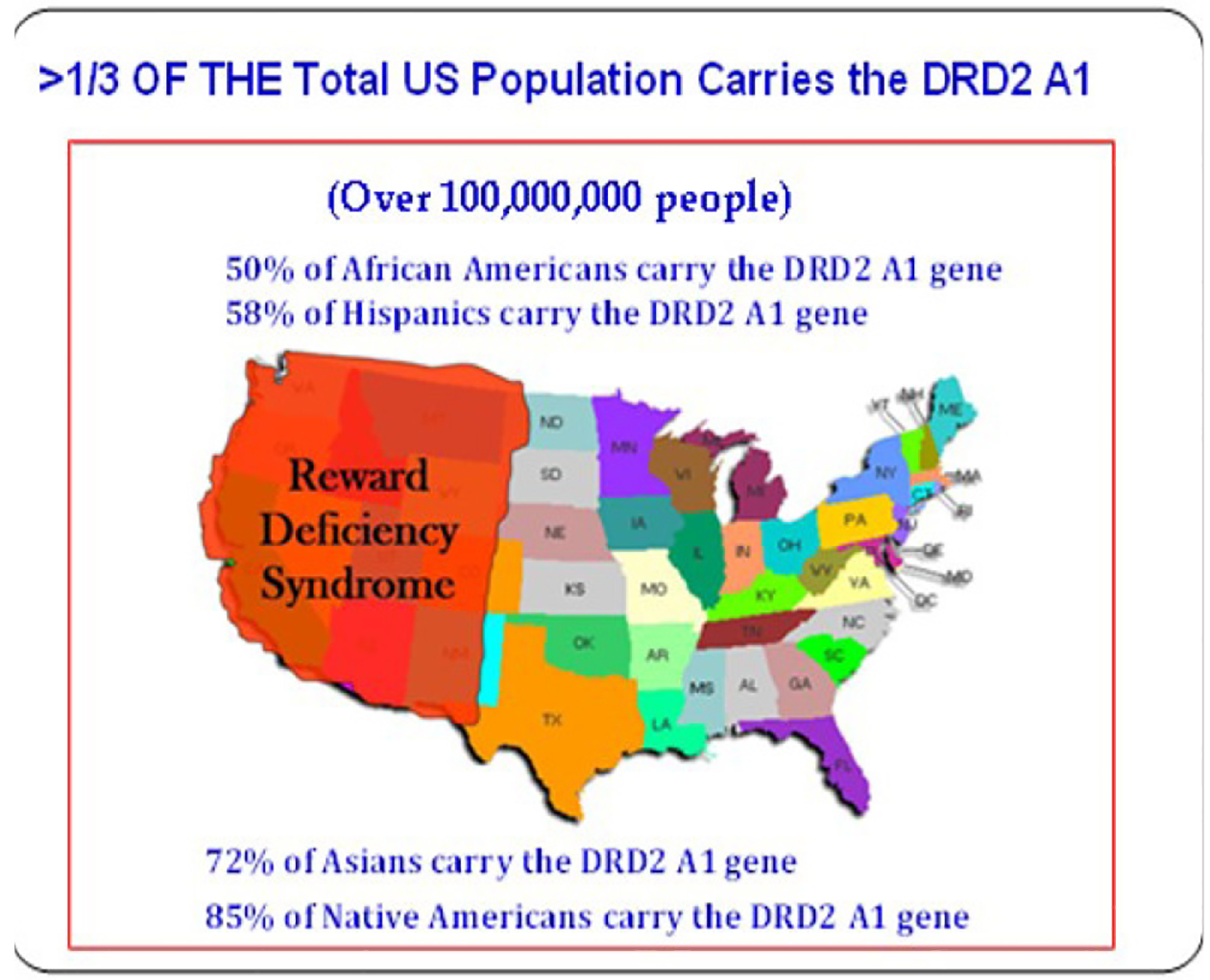
A metaphor showing that RDS in terms of the population of 100,000 million is akin to the entire state of California.

In fact, genetic studies have verified that low dopamine function in the brain attributable in-part to a dopamine D2 variant which is inborne in at least 33 percent of our USA population showing as high as a 40% reduction in D2 receptors in the brain [8] which is akin to the entire population of California (Figure 3). RDS is indeed considered an umbrella term for a remarkable array of shared abarrant behaviors like an octopus [30].

**Figure 4:**
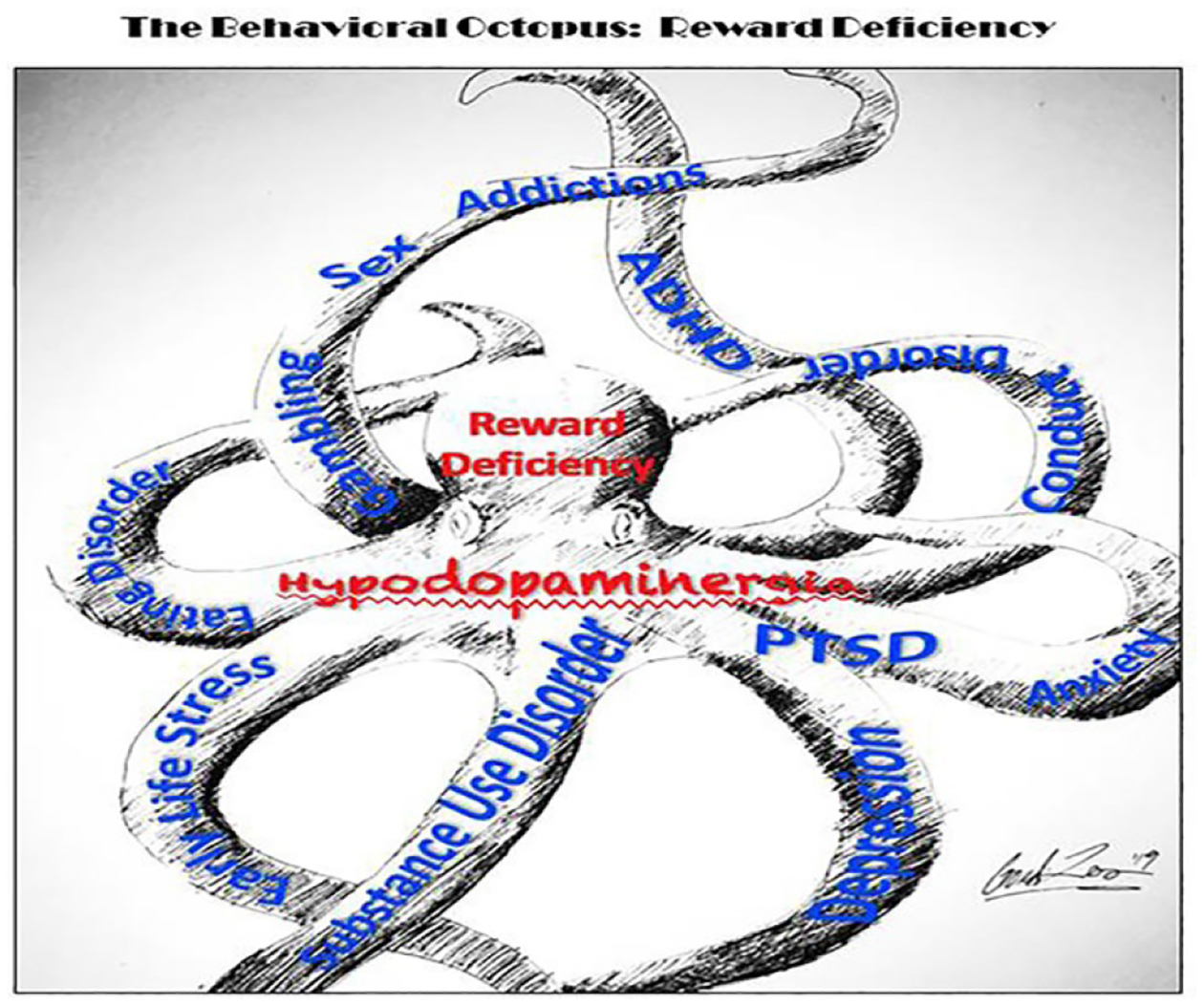
RDS: The behavioral octopus. Schematics show many arms of individual disorders with unique characteristics that share a common foundation of low dopamine signaling tone (hypodopaminergia); a foundational cause/consequence of reward deficiency (original artwork by Steven Gondre-Lewis).

These other behaviors include an array of disorders, such as PTSD, ADHD, Tics, Tourette Syndrome, Autism, Asperger Syndrome, OCD, “compulsive” sexual practices, binge eating and others. The relationships of these disorders become apparent with the understanding of the common genetic factors underlying them (Figure 4).

Carriers of this known DNA antecedent at birth, is that you are more likely to suffer from a whole host of things, that fits within the construct of RDS including OCD, ADHD, PTSD and -- you guessed it – all addictions including drugs (like opioids) exceedingly high doses of THC (as in waxes), booze, gambling -- that reduced dopaminergic receptors (D1-D5) can be a gateway to the underworld of the psyche [31].

**Figure 5:**
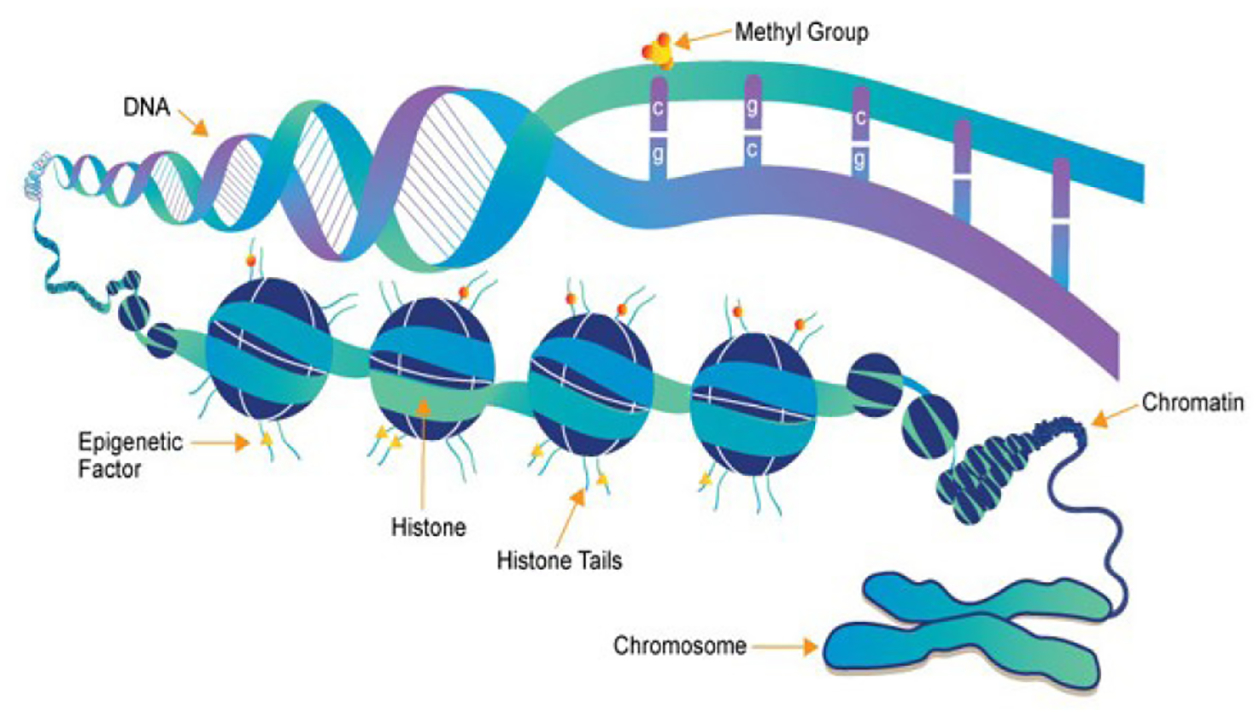
Mechanisms of epigenetics [36].

So, to help us understand this phenomenon, RDS may start in-part with the DRD2 gene (of cause other reward gene induced polymorphic anomalies), which is responsible for growing D2 receptors while we are still in the womb. It undoubtedly could be in many individuals an inherited polymorphism known as the A1 allele. This variant as mentioned earlier, causes an overall reduction in the number of D2 receptors, especially in the brain [8]. So, with a plethora of compelling evidence addiction runs in families, from one generation to another generation, and is in total agreement with the RDS concept [32]. Yes, this is indeed akin to the idea of the importance of nature, now known to be epigenetics. While not excluding nurture, there is overwhelming research demonstrating that addictions shared across generations (like father, like son) correspond with this unwanted dopamine allele [33–35]. In this regard, we consider the formula P = G + E. Whereby, P = Phenotype; G = DNA or Genetics, and E = Environment or epigenetics. Simply, the alleles in general are passed down to the same offspring who end up becoming addicts just like good old dad or mom. However, that is just one part of the addiction prone equation genetically, but what about the environment? There is a new field called “epigenetics” (Figure 5).

### Epigenetics – something that we do not have in our genes, and we can still pass on to our children - one important question is what makes me – “me”.

Are our genetics our destiny? Is the sequencing of DNA the last step in really knowing what we are made of? With the development of revolutionary twentieth century technology that allows us to assess genetics, researchers hoped for the answers to these questions. This enthusiasm has yielded many discoveries, including why we have blue eyes, where albinism comes from and whether each of us can be born with cystic fibrosis. Along this journey was the remarkable 10-year project of learning about the human genome – over 3 billion bricks (the so-called pairs of principles) defining man both as a species, but also as an individual its 30 thousand functional genes.

However, during the last decade scientists realized that discovering genes and linking them with traits (such as diseases and behaviors) is only the first piece of an extremely complex puzzle, which we call ourselves fitting into the species known as *Homo sapiens*. One resounding question to ponder is how identical twins (with identical genomes) differ phenotypically and develop different attributes? A new subfield of biology – epigenetics – comes to the rescue.

Epigenetics deals with genetic alterations that do not result from DNA sequence mutations. This leads to the formation of inherited traits intra-generationally. For example, in a beehive, all bees are genetically identical. Yet, worker bees, soldier bees and queen bees come from the same genome. This occurs due to differences in diet: larvae that receive pollen develop into worker bees, and larvae who receive royal jelly become queens. This phenomenon can be explained by the principles of epigenetics. An important mechanism of epigenetics is DNA methylation, i.e. attaching a small “marker” directly to nucleotides (components – A, T, C, and G) within the DNA. Histone modifications. But what are histones? Histones are proteins necessary to keep the spatial structure of our DNA. The addition of a “marker” to the histone will loosen the DNA structure, potentially reducing genetic expression of the formation of that specific protein (e.g., methylation onto DRD2 receptors decreases the number of D2 receptors formed in the brain) [37]. Moreover, acetylation additions to histone do the opposite and strengthen DNA structure and as such enhance expression of the corresponding transcription protein.

A breakthrough occurred in the field of epigenetics in 2007. Mice with the *Agouti* gene, which plays an important role in yellow color, diabetes, and obesity: causing a rapid increase in death risk, were fed with food as a source of methyl groups (“markers”) during the experiment. To great surprise, the born offspring were brown, slim, healthy, and non-diabetic. The *Agouti* gene, while still present, was silenced by methylation. What is more, this effect was even present in the following generation of mice. One conclusion was reached – offspring can be influenced by the dietary intake of both parents and grandparents. Quite remarkably, it turns out that smoking, moving frequently or even thinking positively is of immense importance to our future generations. It is especially important that we love our pups, as all these actions influence the changes in our epigenome [38].

DNA does not determine our destiny, although it plays a significant role in molding it. It has been previously stated *“Genetics loads weapons, but it is epigenetics that pulls the trigger”*. One interesting experiment from Yasmin Hurds group at Mount Sinai in NYC, showed that when pure THC is injected into mice, two generations later the offspring showed a decrease in D2 receptors and a high increase in heroin ingestion [39]. Specifically, Hurd’s group showed that maternal cannabis use changes the development of mesolimbic D(2)R in offspring. This occurs through epigenetic mechanisms that regulate histone lysine methylation, and the ensuing reduction of D(2)R may impact addiction vulnerability later in life. Additionally [40], alterations of the endogenous opioid system were also found in the brain of adults who were exposed to THC in adolescents. Striatal preproenkephalin mRNA expression was exclusively increased in the NAc shell; the relative elevation of preproenkephalin mRNA in the THC rats was maintained even after heroin self-administration. Moreover, mu opioid receptor (muOR) GTPcoupling increased in mesolimbic and nigrostriatal brainstem regions in animals pretreated with THC. MuOR function in the NAc shell was found to be a correlate to heroin intake. The take home message is that nurture along with nature shapes who we really are [39, 40]. This work agrees with much earlier work in the laboratory of Blum, before we even knew about epigenetics. It was found that prenatal administration of pure THC to pups resulted in a generational alteration of an increased sensitivity to both enkephalin and even norepinephrine using vas deferens [41].

With all this said, the bottom line is that some individuals may have two copies of the A1 variant with possibly 40% fewer D2 receptors in some brain areas like the reward site of the brain called the NAc [42, 43]. In fact, there is real neuroimaging data that actually shows less excitement in goal achievement (Motivation) and reward. In a paper by Eric Stice and his group (Published in the journal *Neuroscience*), carriers of DRD2 A1 variant had a blunted brain response to a milk shake compared to carriers of the normal DRD2 variant. They suggested that this blunted brain response may contribute to obesity because carriers of the risk variant never get satisfied [44]. Compared to most people, the brain-reward response is flattened. Thus, this finding resonates to BB King, *“the thrill is gone”!* However, we must now ask “maybe it was never there at all”. Is that the itch that Gerry had all his life, and could it be that crawling out of his skin is indeed the “preaddiction” or RDS risk just like his father.

If you have the risk variant of the DRD2 gene, which results in decreased D2 receptors, you are more likely to experience RDS. So how are you going to get the thrills those around you seem to get? Well, drugs (including alcohol or even weed of cause), or is it from food or gambling, and other super fun stuff (like sky diving) might be the most effective way. For many people, these rewards are exciting enough. For those of us with a tendency to be under-excited, or brain blunted, seeking out exciting ways such as drug seeking might be one of the few ways to feel engaged, lively, and somewhat “pseudo normal”. It is known, for example, that certain polymorphic genes like the DRD4 increase novelty seeking behavior [45].

While of course there are some unanswered questions and more required intensive research, it does appear that reward deficiency results in individuals seeking more intense dopaminergic reward stimulation. Understanding most of this raises the eye-opening suggestion of the importance of loving your pups. It is also interesting that eating a chocolate bar or riding a roller coaster may for some people be exciting but for others is not that thrilling as BB Kings song – *“the thrill is gone”*. Simply put – pleasure that is commonly experienced by anticipation and approach is decreased with RDS.

In the case of both Pete and Gerry, even without any genetic antecedent it is well-known that their pre-frontal cortex is unmyelinated and as such the decision-making capacity is compromised [46]. Even though because of neurodevelopmental issues we note that teenage thrill seeking seems to be an instinct and as such it is not only inevitable, but studies have shown that teenagers because of neurodevelopment despite the innate low dopamine function in many, most show a high dopamine activity [47]. This translates to why young people find even weed and alcohol to be potent. It is worth-while noting that thrill seeking and drug taking is just part of their everyday lives unfortunately placing them in harm’s way independent of their genetics. This unresolved issue and poor decision-making ability due to unmyelinated pre-frontal cortex, where one’s executive function resides, makes it extremely difficult to reason with our youth up-until age 27 and rehab centers are filled with individuals at a lower age.

While we believe that early genetic at-risk testing even at birth without labeling anyone as an “addict” but with preaddiction risk, could be a crucial step to help parents know what to expect from their children in a world filled with temptation and such dangerous drugs for example opioids laced with Fentanyl killing hundreds of thousand prematurely especially here in America and higher with especially African Americans [48].

As Mark Lewis, a naysayer of the disease model of addiction, reiterated in his piece *“the thrill is gone”* published in Psychology Today in 2013, where he espoused but wondered if RDS would help explain in-part the unforgiving and sometimes fatal romance with psychoactive chemicals. For BB King the thrill was there for a while, but now it’s gone. This simple statement rings true for many, but was the thrill ever there to begin with? It is this kind of questioning that resonates with the idea of awareness of our genomes, epigenetics, and ways to assist in its attenuation prior to the unwanted sequalae of RDS and all its components. Foojan Zeine has developed Awareness Integration Theory (AIT), and Nicole Jafari and their team have done extensive research on the efficacy of this tool. AIT is a multimodal and holistic approach utilized in addiction treatment that addresses addiction’s physical, emotional, mental, and spiritual aspects. AIT is based on the belief that addiction is a symptom of deeper issues, such as trauma, depression, anxiety, and adverse childhood experiences. AIT treatment typically includes individual therapy, group therapy, and family therapy. The AIT 6 phase intervention addresses the awareness of thoughts, feelings, behaviors, and the impact of addiction on different areas of life. AIT fosters the integration of the split parts through trauma healing exercises and promotes responsibility and accountability toward self-growth, healing, and creation of healthy relationships with others [49].

In response to the drug abuse and behavioral addiction crisis affecting so many, Blum’s group have developed a genetic addiction risk severity (GARS^®^) test that measures ten reward genes that includes major neurotransmitter systems such as serotonin, opioid receptor (e.g. Mu), endorphins, GABA, glutamine, dopamine, and catabolic enzymes (e.g., COMT and MAO-A). Since its inception in 2014, this domestic and foreign patented test is called GARS with 84 papers listed (December 8, 2023) in the literature [50]. Secondly, since 1976, the same group have developed a highly effective nutraceutical complex (KB220) that neuroimaging shows “dopamine balance” even in abstinent heroin dependent patents. To date there are north of 35 clinical trials that show positive clinical outcomes along with several animal studies demonstrating mechanisms of action [51].

**Figure 6:**
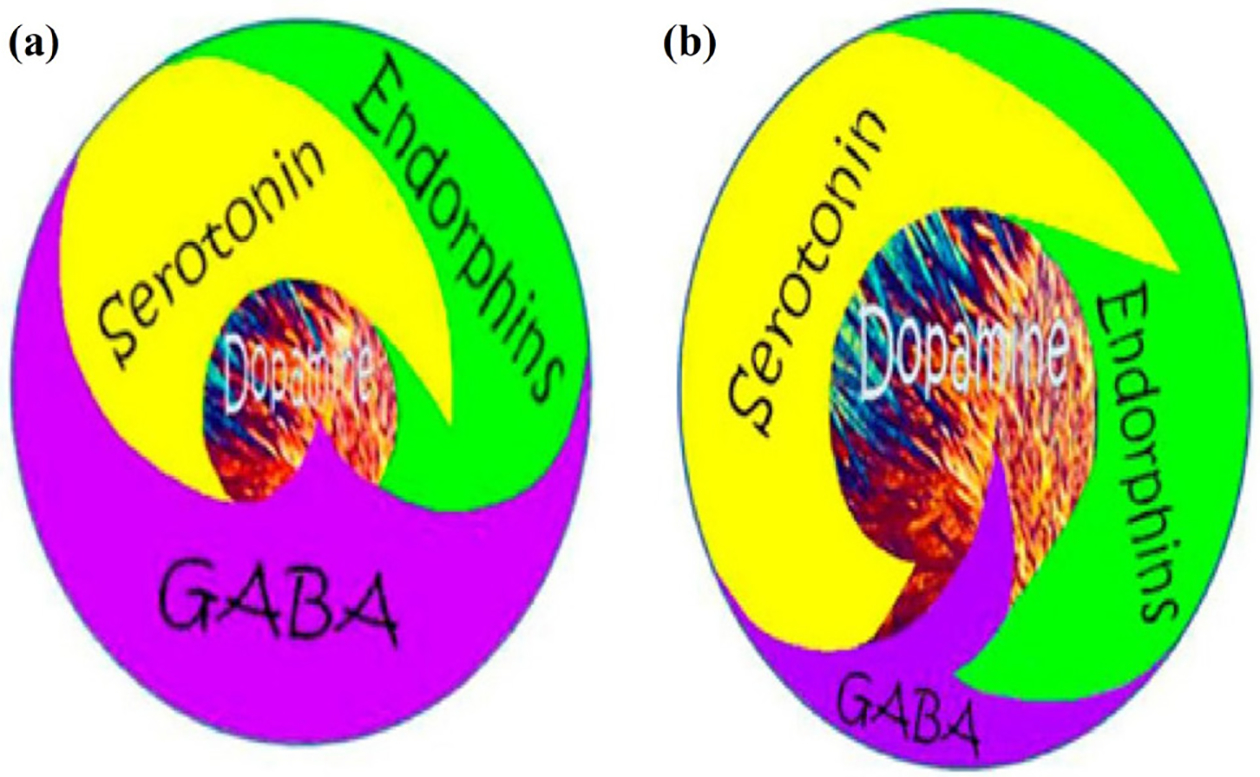
Schematic representation of Brain Reward Cascade. **(a)** Abnormal unbalanced neurotransmission showing high GABA transmission with reduced dopamine release: Unhappy Brain. **(b)** Normal balanced neurotransmission showing appropriate amount of dopamine release: Happy Brain feeling of well-being [26].

The basic idea of this complex construct is to bring about a “Happy Brain” with just the right amount of dopamine released continually at the reward site (N. Accumbens) converting a “Unhappy Brain” even starting at birth (Figure 6) via finding ways to promote dopamine homeostasis.

**Figure 7:**
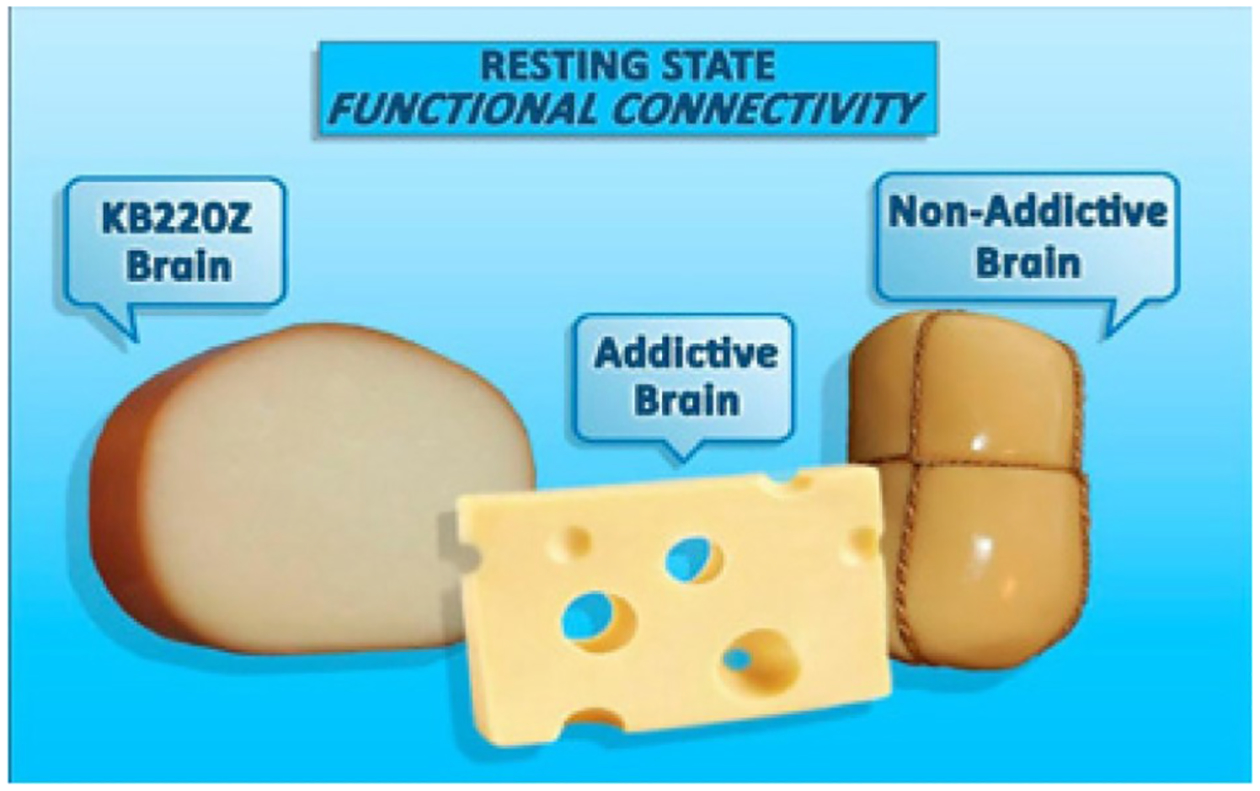
Functioning brain connections represented by cheese. Addictive Brain: lacks connectivity at rest represented by holes (no crosstalk) compared to Non-Addictive Brain. KB220Z helps restore resting state functional connectivity and consequentially better decision making (with permission from Blum et al.).

Recently neuroimaging studies have uncovered reduced resting state functional connectivity (rsFC) as a culprit in addictive behaviors [52]. “Normal” rsFC can be understood as “cross talk” meaning that different parts of the brain communicate, for example, the hippocampus (Memory) talks with the accumbens (Craving) talks with the Cingulated Gyrus (Decision-making). Reduced functional connectivity at rest puts the individual at risk for addictive-like behaviors-view the cartoon of Swiss cheese (Figure 7).

**Figure 8:**
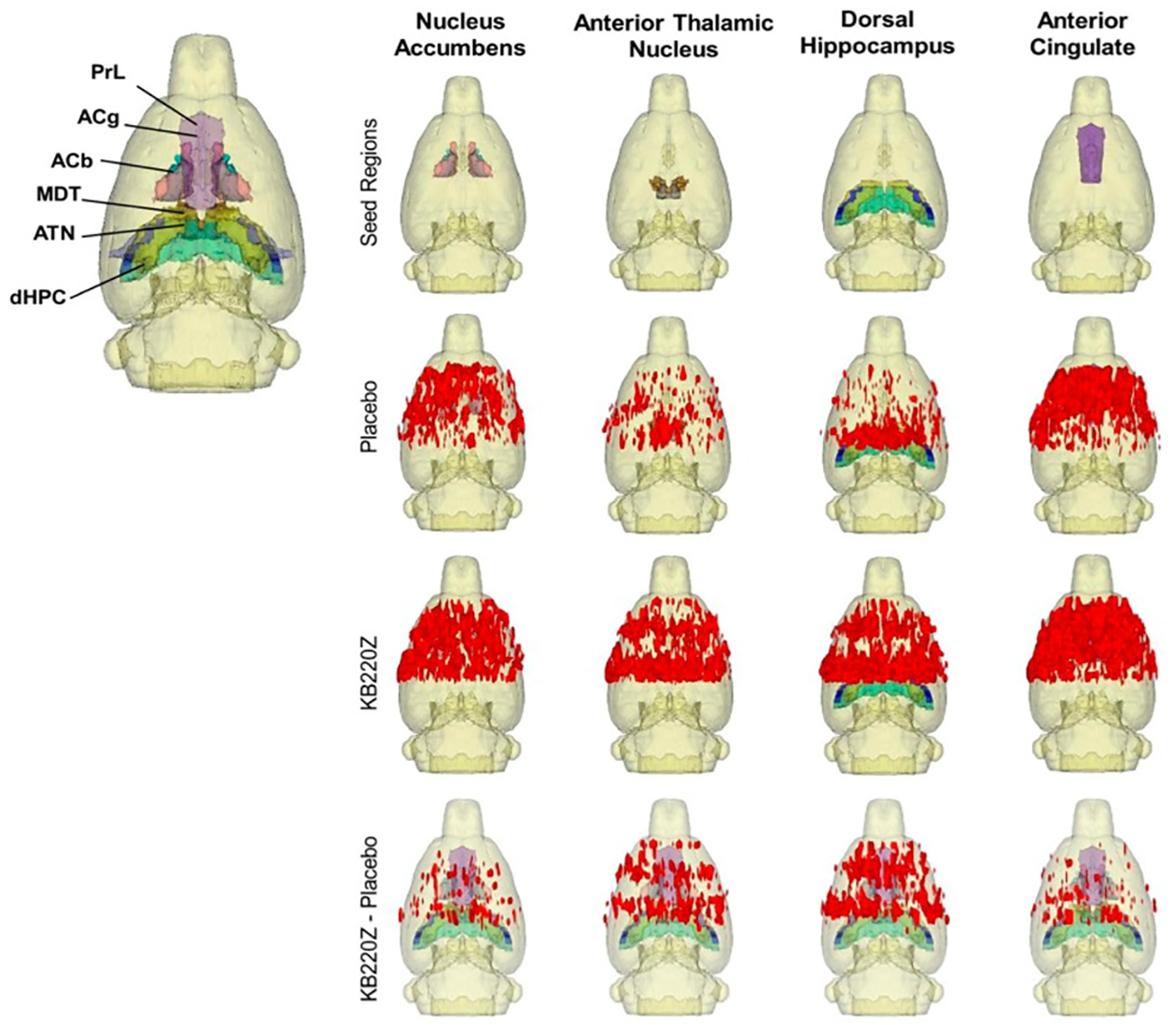
Composite 3D functional connectivity maps comparing KB220Z and placebo. The top row shows the segmented 3D ROI used as seed for the placebo and KB220Z maps seen below them. High clustering of voxels occurs within the seed regions for both placebo and KB220Z groups. Greater connectivity based on the number of voxels showing high correlation coefficient values is observed in the KB220Z maps. Difference maps (KB220Z minus placebo) are shown in the bottom row. Maps are set at a lower statistical threshold of p < 0.005 (voxel cluster size corrected) (with permission from Febo et al.).

Published work from Marcelo Febo’s laboratory with non- addicted rats, KB220Z was shown to activate connectivity in brain regions associated with memory, decision-making and craving compared to placebo controls. These anatomical regions include the prelimbic and infralimbic loci, the NAc, the cingulate gyrus, anterior thalamic nuclei, and the hippocampus (Figure 8). KB220 may induce positive epigenetic changes (like increases in rsFC) to counterbalance the negative epigenetic insults due to drug toxicity and stress (like reduced rsFC) [53].

**Figure 9:**
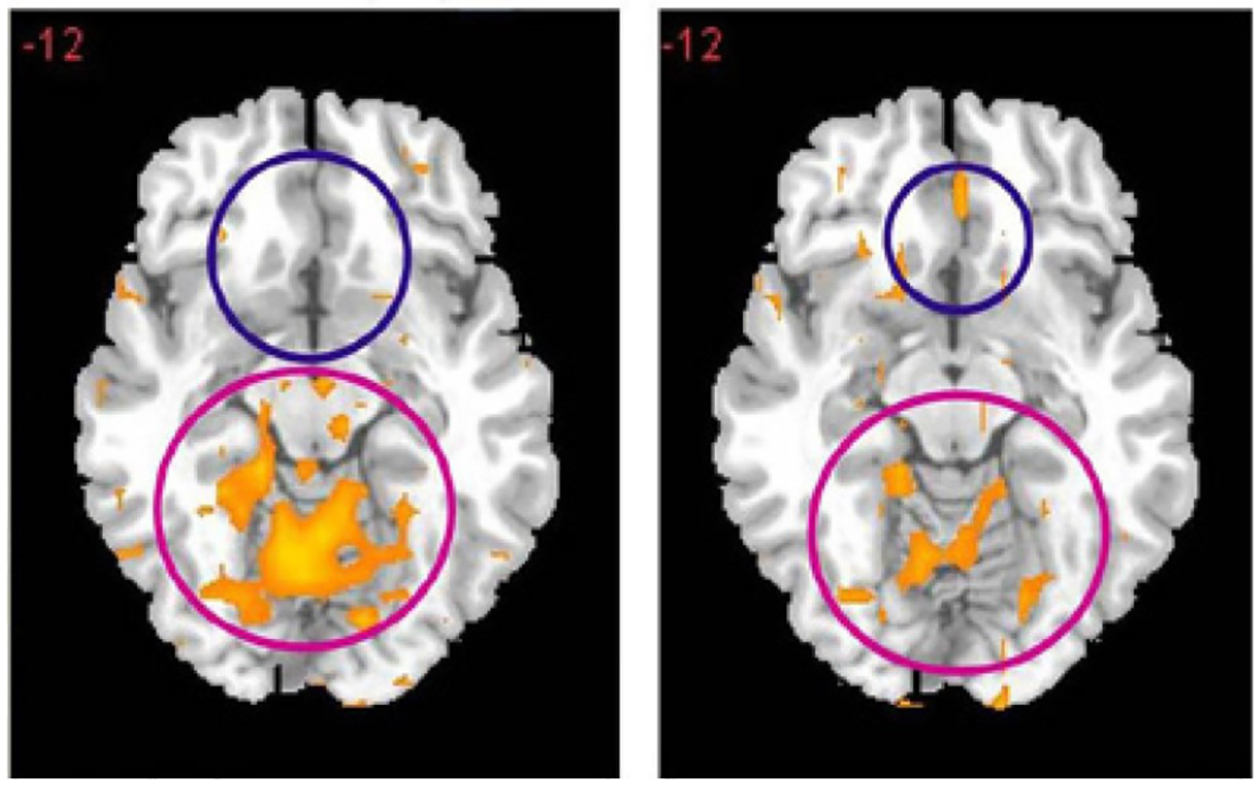
Resting-state fMRI 1 hour after one dose KB220Z. Placebo left side vs KB220Z right side (with permission from Blum et al.).

Finally, in a placebo-controlled crossover study, rsFC was significantly restored in abstinent heroin addicts given KB220z. One-hour after being given oral KB220Z, dopaminergic pathways were activated, heightened emotionality seen as hyper dopaminergic activity in the cerebellum is reduced and rsFC was restored. This might show “dopamine balance looks like (Figure 9) [54]. Similar results have been found in abstinent psychostimulant abusers [55].

## Discussion

There is still much to be done including even gene editing, but now we have the right questions to ask, and the lay public can appreciate the extensive research from many laboratories across the globe embracing the RDS concept involving both DNA preaddiction risk at birth and environmental or epigenetic impact from grandparents to parents to children [56].

While the jury is still out regarding the exact causes of RDS including not only genes but psychological aspects as well the role of spirituality and even something called “Geneospirituality” [57] even the important pre-testing children for brain health as espoused by Braverman and others. Nevertheless, we are poised with a message of hope and our governmental research bodies are performing highly sophisticated research in multiple disciplines. As Dr. Nora Volkow has strongly espoused, we need “ALL HANDS-ON DECK!”.

Every day, there are several millions of people that are increasingly unable to combat their frustrating and even fatal romance with getting high and/or experiencing “normal” feelings of well-being. What price should they pay to experience this innate natural experience? People have been thrown into jails in Texas and other states, for just smoking weed, but many are seeing the day of light because of pardons. While global societies should not go back to such harsh punishment for getting high, we must be aware of the dangers that are occurring across the legalized states regarding marijuana. The major difference is the very high uncontrolled percentages being sold under the label of governmental legalization [58–64]. Finally, it is our opinion that since it is well known that powerful pharmaceuticals, especially to treat mental illness induces many neurological modifications or adaptations, many negative, there seems to be a need for innovative thinking. This could include novel non-addicting and non-invasive electro therapeutic stimulation (Personalized (P) Transmagnetic Stimulation [PrTMS] as deeloped by Kevin Murphy’s group, H-WAVE (for pain), neuroimmune agents, pro-dopamine regulators, exercise, even deep brain stimulation and gene therapy [65–108].

As of 2021, opioid overdoses account for 100,000 premature deaths. Underreported comorbidities of reward dysregulation due to genetic antecedents and epigenetic insults include neuropsychiatric and cognitive impairments. Genome-wide association studies found substance use disorder and depression are frequent comorbidities, and significant correlation with NEGR1 expression in the hypothalamus and DRD2 in the NAc. There remains no brain health assessment standards despite the rise in SUD and neuropsychiatric illness [21].

A proposed standard objective Brain Health Check (BHC) requires extensive data available to treat clinical syndromes in psychiatric patients [109]. A BHC would consist of the following domains: memory, attention, neuropsychiatry, coaching, and neurological imaging. This methodology would be reliable, accurate, and cost-effective. Based on a review by Braverman et al. [109], the following assessments of Memory, Attention, Psychiatric, and Neurological imaging are recommended for use in the BHC: Central Nervous System Vital Signs (Memory), Test of Variables of Attention (Attention), Millon Clinical Multiaxial Inventory III (Neuropsychiatric), and Quantitative Electroencephalogram/P300/Evoked Potential (Neurological Imaging). Finally, the “Brain Reward” consortium suggests continuing the collection of data for a new standard BHC in addition qEEG/P300/Evoked Potentials and genetically guided precision management of “dopamine homeostasis” for the purpose of diagnosing and treating RDS, in an effort to prevent the consequences of dopamine dysregulation epigenetics passing on to our children, our children’s children, and all future generations.

## Limitations

While we have tried to develop a thoughtful argument for espousing the notion that potentially people are getting high because the thrill is gone and provided some molecular neurobiological aspects to help support this idea, there are still many questions that need to be researched. Moreover, this is not a systematic review of the literature and as such many articles have been cherry picked to support our concept. This approach therefore is somewhat biased, and we encourage the scientific community to not only engage in original research to confirm or refute these constructs, as well as carefully executed systematic reviews.

## Recommendations

Based on the information provided and discussion thereof, the following treatment options are recommended:

- It is suggested that internists, family medicine practitioners, and psychiatrists evaluate and screen patients for drug use, abuse, or dependence.
- Brain screening should be offered to appropriately assess medication management and possibly behavioral modification.
- Genetic screening, such as GARS testing, may be utilized to identify DNA risk antecedents in patients and their families.
- Inpatient detox facilities may be necessary for detoxification, or outpatient detox may be achieved through the use of methadone or suboxone for a short time-period followed by more gentle pro-dopamine regulation, such as KB220 variants.
- Patients may benefit from residing in a sober living home for 3 – 12 months, depending on their needs, to change their environment from habitual drug use at home. Patients can grow and learn from group dynamics at sober living homes with recovery programs.
- Outpatient detox and medication management of mood disorders, anxiety disorders, or drug-induced psychotic disorders should be under the care of a psychiatrist with expertise in the field of addiction.
- Patients may benefit from weekly individual psychotherapy with an addiction expert certified in the Awareness Integration Theory to work on underlying emotional traumas and intrapersonal and interpersonal dynamics.
- Weekly individual counseling with a chemical dependency counselor can help patients work on day-to-day addictive behavioral modification.
- Patients should attend and participate in 12-step programs and obtain a sponsor or enroll in an outpatient group program.
- Family members should also participate in 12-step programs.
- Long-term recovery groups should include a relapse prevention program utilizing telephonic programs.
- Maintenance therapy utilizing safe, non-addictive, and non-invasive modalities such as Personalized Transcranial magnetic stimulation (for many RDS behaviors including depression and anxiety), H-wave device for pain control, and pro-dopamine regulation for induction of dopamine homeostasis is recommended.
- Pain clinics should rigorously adhere to CDC guidelines.
- There should be an increase in research funding for both basic science (animal models of RDS) and clinical science.

## Conclusion

The real question to ponder is there a “cure” for this unwanted molecular genetic rearrangement at birth impacted by the environment past onto at least two generations via epigenetic effects inducing impaired brain dopamine impacted by many other neurotransmitters as observed in RDS. In the near or even distant future through the remarkable dedication of worldwide scientific exploration it is our hope that Earth’s inhabitants will be free of pain and disease and find paradise and reward as denoted in the “Garden of Eden!”.
